# Pancreatic cancer cells spectral library by DIA-MS and the phenotype analysis of gemcitabine sensitivity

**DOI:** 10.1038/s41597-022-01407-1

**Published:** 2022-06-09

**Authors:** Ran Kong, Xiaohong Qian, Wantao Ying

**Affiliations:** State Key Laboratory of Proteomics, Beijing Proteome Research Center, National Center for Protein Sciences (Beijing), Beijing Institute of Lifeomics, Beijing, China

**Keywords:** Proteomics, Tumour biomarkers

## Abstract

Data-independent acquisition (DIA)-mass spectrometry (MS)-based proteome strategies are increasingly used for detecting and validating protein biomarkers and therapeutic targets. Here, based on an in-depth proteome analysis of seven pancreatic cancer cell lines, we built a pancreas-specific mass spectrum library containing 10633 protein groups and 184551 peptides. The proteome difference among the seven pancreatic cancer cells was significant, especially for the divergent expression of proteins related to epithelial-mesenchymal transition (EMT). The spectra library was applied to explore the proteome difference of PANC-1 and BxPC-3 cells upon gemcitabine (GEM) treatment, and potential GEM targets were identified. The cytotoxicity test and GEM target analysis found that HPAC, CFPAC-1, and BxPC-3 were sensitive to GEM treatment, whereas PANC-1 and AsPC-1 were resistant. Finally, we found EMT was significant for CFPAC-1, AsPC-1, and PANC-1 cells, whereas BxPC-3 and HPAC cells showed more typical epithelial features. This library provides a valuable resource for in-depth proteomic analysis on pancreatic cancer cell lines, meeting the urgent demands for cell line-dependent protein differences and targeted drug analysis.

## Abstract (summary)

**Table:** 

Measurement(s)	protein expression profiling
Technology Type(s)	Mass Spectrometry

## Background & Summary

The average 5-year survival rate of pancreatic cancer is 8%, rendering it the fourth leading cause of cancer-related deaths worldwide. It is expected to become the second leading cause of cancer-related deaths by 2030^1,2^. Pancreatic ductal adenocarcinoma (PDAC) accounts for > 90% of all pancreatic malignancies^3^. Asymptomatic early stage and lack of reliable early diagnostic biomarkers hinder pancreatic cancer detection before it has spread to other organs, and the insensitivity of pancreatic cancer to chemotherapy and radiotherapy leads to a high mortality rate^4^. Therefore, understanding the biology and mechanism of PDAC progression and metastasis is essential to improve early detection and treatment. The rapid development of proteomics and chemical biology technologies has recently stimulated interest in elucidating the complex mechanisms underlying pancreatic cancer.

The study of protein composition in pancreatic cancer is important for discovering new strategies for early diagnosis. The interaction between cancer cells and the host is reflected in the constantly changing protein composition during carcinogenesis and metastasis. Studies based on transcriptome and genome sequencing have identified four major driving genes in pancreatic cancer: the oncogene KRAS (mutation rate greater than 95%), tumour suppressor gene TP53 (50%–75%), CD-KN2A/p16, and SMAD4 (mutation rate > 90%)^5,6^. However, these analyses failed to provide information regarding post-transcriptional regulation, post-translational modification, or protein localisation.

Data-independent acquisition (DIA), a label-free quantitative proteomics approach, is a new MS technology developed in recent years. The entire scanning range of the mass spectrometer was divided into several windows, and all precursor ions in each window were selected, fragmented, and detected cyclically at high speeds, and all the fragment information of all the precursor ions in the sample were recorded without any bias or omission^7^. With a much lower level of missing values, DIA provides quantification accuracy and repeatability, achieving high stability and high-precision, rendering DIA-MS extremely suitable for proteome researchers with large sample sizes. DIA has been successfully applied in disease proteomic research such as Parkinson’s disease, Alzheimer’s disease, and liver cancer^8–10^.

Presently, nucleoside analogue gemcitabine (GEM) monotherapy or combinational therapy has been the treatment of choice for advanced pancreatic cancer, doubling the 5-year survival rate of around 16%-21% of patients^11–14^. However, after a few weeks of treatment, tumour cells originally sensitive to GEM develop drug resistance. GEM resistance is one of the most significant factors affecting the efficacy of chemotherapy, leading to poor prognosis^15,16^. GEM resistance is closely related to the tumour microenvironment, extracellular matrix, and intracellular signal molecule regulation, which are decisive factors affecting PDAC treatment. Activation of epithelial-mesenchymal transition (EMT) is associated with the invasion and metastasis of pancreatic cancer and resistance to GEM chemotherapy^17–19^. Although the transformation from epithelial to mesenchymal cells is achieved through TGF-β, HIF-1α, WNT, NF-κB, and other mediated signal pathway expressions, its downstream molecular signal has not been determined^20–22^.

In this study, we first used the combination of peptide pre-fractionation with DDA tandem MS strategy to acquire MS2 spectra for seven pancreatic cancer cell lines. Which, together with the spiked indexed Retention Time (iRT) peptides information, were imported Spectronaut ™ (version 15.0) software to establish a pancreatic cancer cell-specific spectra library for DIA-based proteome application. Then, based on the established DIA pancreatic cancer cell spectrum library, we analysed the proteomics of PANC-1 and BxPC-3 cells before and after GEM treatment. In addition, the Cell Counting Kit-8 (CCK-8) kit was used to detect the half-maximal inhibition rate of pancreatic cancer cells. Finally, based on the known and potential targets of GEM- and EMT-related biomarkers, and the analysis of the relative expression levels of proteins in the seven pancreatic cancer cell lines, we determined the GEM-sensitive and EMT phenotypes of the cell lines. These data provide a supportive resource for in-depth proteomic analyses of pancreatic diseases.

## Methods

### Study design

**Fig. 1:**
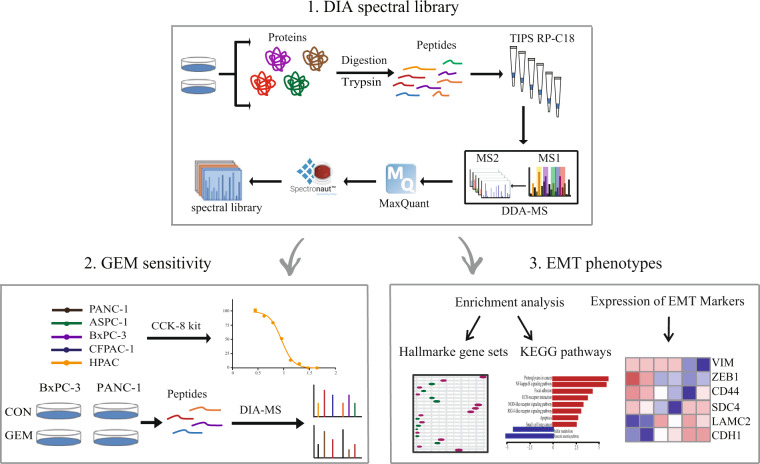
The workflow of the study. 1. Generation of DIA spectral library of pancreatic cancer cells. 2. Sensitivity of GEM in cell lines. 3.The EMT phenotypes of pancreatic cancer cell lines.

Seven commonly used human pancreatic cancer cell lines were selected to generate the spectral library: the pancreatic cancer cell lines AsPC-1, BxPC-3, CFPAC-1, HPAC, and PANC-1; human pancreatic stellate cells HPaSteC (HPSC); normal human pancreatic ductal epithelial cells HPDE6-C7 (H6C7). Eighty-four original MS data files were obtained from the pre-fractioned peptide by basic reversed-phase chromatography (Fig. 1). We investigate the effect of GEM on pancreatic cancer cell lines using a CCK-8 kit. We then analysed the proteomics of PANC-1 and BxPC-3 cells before and after GEM treatment using the DIA strategy based on the spectrum library. A total of 77928 unique peptides and 7089 corresponding proteins were identified.

### Cell samples

We selected seven commonly studied human pancreatic cancer cell lines grown under the same conditions: five pancreatic cancer cell lines, including AsPC-1, derived from cancerous ascites produced by xenotransplantation of human pancreatic cancer in nude mice and can express carcinoembryonic antigen, human pancreas-associated antigen, human pancreas-specific antigen, and mucin^23^; BxPC-3, an adenocarcinoma cell line, derived from the pancreatic body^24^; CFPAC-1, derived from a liver metastasis of cystic fibrosis (CF) in a 26-year-old white male^25^; PANC1, derived from the pancreatic ducts of patients with epithelioid carcinoma^26^; HPAC, a cell line established by xenotransplantation of primary human pancreatic ductal adenocarcinoma^27^; a pancreatic stellate cell line, HPSC, derived from pancreatic exocrine myofibroblasts, is responsible for synthesising and degrading extracellular matrix components and promoting tissue repair^28^; a normal human pancreatic duct epithelial cell line, H6C7, which can be used as a model for studying human pancreatic ductal cell carcinogenesis^29^. The basic characteristics of pancreatic cancer cell lines are shown in Fig. s3a. Cells were cultured in Dulbecco’s modified Eagle medium (DMEM) containing 10% fetal bovine serum (FBS). When cells grew to 80% confluence in the logarithmic growth phase, they were washed with ice-cold phosphate-buffered saline (PBS) three times, and the cell precipitate was collected.

### Protein extraction and digestion

Pancreatic cancer cell samples were lysed with T-PER® and sonicated on ice (99 cycles; 200 W, 1 s on and 2 s off). After incubating on ice and cracking for 10 min, the lysate was centrifuged at 14,000 × g for 15 min. The supernatant was recovered as an extract for subsequent analyses, and quantified using the Bradford assay. The protein was digested using the filter-aided sample preparation (FASP) method^30^.

### Pre-fractionation of peptides

For each cell line, 50 ug of peptides were pre-fractionated in a tip filled with C18 media, by a sequential elution using 90 µL of solution containing 6%, 9%, 12%, 15%, 18%, 21%, 25%, 30%, 35%, and 50% acetonitrile in ammonia water at pH 10, respectively. The 7th, 8th and 9th fractions were merged into 1st, 2nd and 3rd respectively, and the 10th into 6th. Finally, the fractions were collected and combined into 6 fractions.

### Spectra acquisition by DDA

LC-MS/MS identification of the peptide fractions was performed on an Easy-nLC 1000 System nanoflow high-performance liquid chromatograph (HPLC) coupled to a Q-Exactive HF mass spectrometer (Thermo Fisher Scientific, USA). A self-packed column of 30 cm length (inner diameter 150 μm, ReproSil-Pur C18-AQ, 1.9 μm; Dr. Maisch, GmbH, Tübingen, Germany) was used. The chromatographic conditions were as follows: gradient elution at a flow rate of 350 nL/min (mobile phase A is 0.1% FA aqueous solution, mobile phase B is 0.1% FA-99.9% ACN), 135-min gradient elution: 0 min, 6% B; 0–13 min, 6%–10% B; 13–99 min, 10%–23% B; 99–120 min, 23%–33% B; 120–123 min, 33%–90% B; 123–135 min, 90% B. The ion scan range of the primary mass spectrometer was 375–1400 m/z with a resolution of 120,000.

### iRT-standardised peptide

iRT (Biognosys, Schlieren, Switzerland) was added to all samples as an internal standard before MS injection according to the manufacturer’s instructions.

### DIA analysis

Following the digestion of PANC-1 and BxPC-3 cells before and after GEM intervention, they were identified using LC-MS/MS. The scanning range of MS1 was 400–1200 m/z, followed by MS2, which cyclically selected, fragmented, and detected all ions in each window (25 m/z isolation width, 1 m/z overlap). All experiments were repeated twice, and the iRT-standardised peptide was added for calibration.

### CCK-8 assay

CCK-8 (Dojindo Molecular Technologies, Inc., Kumamoto, Japan) was used to detect cell proliferation. The pancreatic cancer cell lines AsPC-1, BxPC-3, CFPAC-1, HPAC and PANC-1 were inoculated into the wells of a 96-well-plate at 5000 cells per well. After 24 h of incubation, different concentrations of GEM were added, and the cells were further incubated for 48 h. Then, 10 μL of CCK-8 was added to each well, incubated for 2 h, and the absorbance at 450 nm was measured using a microplate reader. Each concentration was repeated four times.

### Data analysis

Qualitative and quantitative proteomic analyses were performed on RAW files obtained by Q-Exactive HF mass spectrometer analysis^31^. We used MaxQuant software (version 1.6.2.3) based on the human Uniprot protein sequence database (version 20200911; 20,375 sequences) containing iRT peptide sequences. The parameter settings of the MaxQuant software were: the restriction enzyme digestion method, trypsin; the missed cleavage site of the enzyme was set to 2, the false positive rate (FDR) of the peptide and protein was less than 1%, and the corrected precursor ion error was set to 5 ppm. The “msms.txt” file output by MaxQuant was imported into Spectronaut™ (version 15.0).

### Gene and protein ID conversions

Bioinformatic analysis was performed on R (version 3.5.4). Different analysis methods and software may require different gene/protein identifiers. We used clusterProfiler and org.Hs.eg.db for conversions of identifiers. Use the official gene symbols approved by the HUGO Gene Nomenclature Committee and the protein identifier using UniProtKB accession number.

### Differential analysis

Differentially expressed protein analyses were done using the “limma” software package (version 3.24.15), and the quantile normalisation method in the “limma” corrected the proteome data^32,33^. After correction, most analyses require the log_2_ logarithmic transformation of the proteomic data for subsequent analysis and filling in the minimum value for the missing values that cannot be processed.

### Enrichment analysis

Gene set analysis was performed using the “GSEABase” and “enrichplot” R package. The biological signal pathways selected for analysis were downloaded from the MSigDB database (Version 7.4, http://software.broadinstitute.org/gsea/msigdb/index.jsp), including 50 cancer hallmark gene sets, 186 Kyoto Encyclopaedia of Genes and Genomes (KEGG) signal pathways, and 7481 gene ontology (GO) biological process ontology’s signal pathways. Based on the above signal pathway entries, differentially expressed proteins were analysed for pathway enrichment. Only enriched signal pathways with false discovery rate-adjusted p-value (p.FDR) < 0.05 were considered.

## Data Records

The raw mass spectrometry (MS) DDA files for library generation and the search results (pepXML) have been deposited to the ProteomeXchange Consortium via the PRIDE^34^ partner repository with the dataset identifier PXD031028^35^.

Besides, the raw MS DIA files of BxPC-3 and PANC-1 cell lines used to discover potential targets of GEM and the DIA spectral library have been deposited to the ProteomeXchange Consortium via the PRIDE partner repository with the dataset identifier PXD030948^36^. The spectral library was labelled as “PC library.xls”. The DIA data analysed in Spectronaut (xlsx) is available from ProteomeXchange Consortium via the PRIDE partner repository with the dataset identifier PXD032263^37^.

## Technical Validation

### DIA spectral library for pancreatic cancer cells

**Fig. 2:**
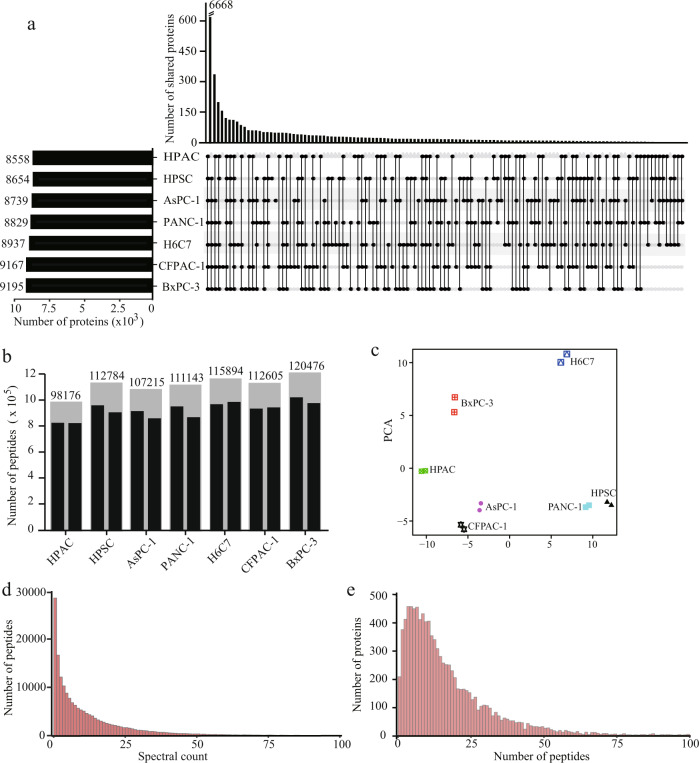
Building of the pancreas-specific spectral library for the DIA strategy. (**a**) Visualisation of intersections of the proteomes using UpSet. (**b**) The number of peptides for each cell line. (**c**) PCA results for pancreatic cancer cell lines. (**d**) The distribution of the number of matched spectra for each peptide. (**e**) The distribution of numbers of matched peptides for each protein.

Seven common human pancreatic cancer cell lines that grew normally under the same conditions were selected to generate the DIA spectral library. A total of 42 peptide fractions was obtained by pre-separating the peptide mixtures, and 84 original MS data files were collected from 84 MS injections. iRT was added to each sample to ensure accurate retention time calibration for better quality control. To evaluate the reliability of the proteomics data, we checked the original MS/MS data and found that the average number of matched spectra for each peptide was 15, indicating that the proteomics data were highly reliable at the peptide level (Fig. 2d). In addition, we found that the average number of matched peptides in the 10,544 proteins was 18 (Fig. 2e).

The MS/MS matching results were used as input to build the spectral library by Spectronaut^TM^, included 10663 proteome groups, 184551 peptides, 283077 precursors, and 2013590 fragments. This resource supports the reliable detection and quantification of 52.28% of all human proteins annotated using UniProtKB, currently the most comprehensive pancreatic cancer-specific DIA spectral library. This method is expected to be widely used in basic research and large-scale proteomics of clinical samples. We then counted the number of peptides and protein groups for each of the seven cell lines and found 6668 proteins in all cell lines (Fig. 2a,b). Principal component analysis (PCA) showed significant differences among the proteome of different cell lines (Fig. 2c). Furthermore, the R package clusterProfiler was used to investigate the results of GO enrichment analysis, revealing that diverse molecular functions were enriched (Fig. s1a–c).

### Biological differences between different pancreatic cancer cell lines in the library

**Fig. 3:**
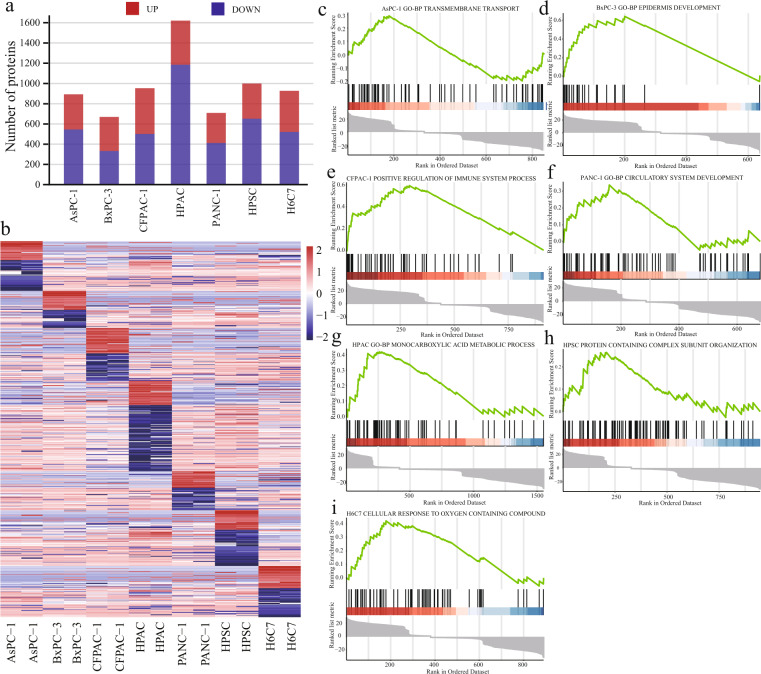
Biological differences among the proteome of different pancreatic cancer cell lines. (**a**) The number of differentially expressed proteins in the cell lines. (**b**) Heatmap view of the up- or downregulated proteins in the seven cell lines. (**c**–**i**) GSEA results in seven datasets.

To determine the type of protein enrichment in each pancreatic cancer cell line, the R language “limma” software package was used to statistically analyse the protein expression profiles of each cell line, and seven clusters were identified. The expression levels of 892, 669, 953, 1621, 709, 999, and 928 proteins showed significant difference for AsPC-1, BxPC-3, CFPAC-1, HPAC, PANC-1, HPSC and H6C7, respectively (log_2_ FC = 2, p < 0.05) (Fig. 3a,b).

We performed gene set enrichment analysis (GSEA) in each of these seven datasets to determine their biological functions. A total of 36, 55, 297, 126, 48, 83, 110 pathways were significantly enriched or depleted in the AsPC-1, BxPC-3, CFPAC-1, HPAC, PANC-1, HPSC, and H6C7 cell lines, respectively. The proteins specifically expressed by AsPC-1 mainly mediated regulation of histone modification, transmembrane transport, and cell adhesion (Fig. 3c). The enriched processes for BxPC-3 cells were mainly involved in epithelial cell development, proliferation, and leukocyte-mediated immunity (Fig. 3d). In CFPAC-1, biological processes such as positive regulation of immune response, DNA replication, and repair were enriched; however, further research is warranted (Fig. 3e). The proteins specifically expressed by HPAC mainly mediated biological processes such as regulation of cell cycle process, monocarboxylic acid metabolic process, and fatty acid metabolic process (Fig. 3g). PANC-1 showed significantly higher expression levels of proteins involved in the circulatory system development, epithelial cell differentiation, and positive regulation of cell adhesion (Fig. 3f). The proteins with significantly higher expression levels in HPSC included those involved in positive regulation of intracellular signal transduction, protein containing complex subunit organisation, and immune response-related cell activation (Fig. 3h). Proteins of higher abundance in the H6C7 cell line included those participating in the cellular response to oxygen-containing compounds, cytoskeleton organisation, and DNA metabolic process (Fig. 3i). We revealed the biological differences between the seven cell lines using proteomic expression profile data, which can serve as a basis for hypothesis-driven discovery or validation research on pancreatic diseases. At present, some relevant treatments based on the molecular biology of pancreatic cancer have not achieved satisfactory results, partly because of the heterogeneity of the tumour, and the difference between *in vivo* and *in vitro* tests has increased its complexity. Therefore, stratifying pancreatic cancer according to heterogeneity and developing a more accurate GEM sensitisation programme should be the focus of future research and development.

### Gene set enrichment analysis of pancreatic cancer cell lines

**Fig. 4:**
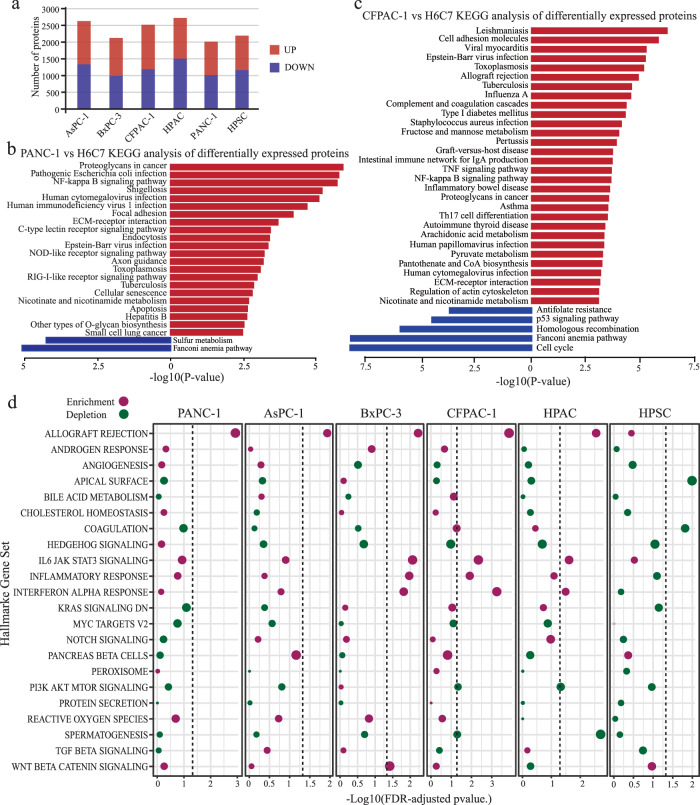
Gene set enrichment analysis of pancreatic cancer cell lines. (**a**) The number of differential proteins in cancer cells compared with normal pancreatic cells. (**b**) PANC-1 vs H6C7 KEGG analysis of differentially expressed proteins. (**c**) CFPAC-1 vs H6C7 KEGG analysis of differentially expressed proteins. (**d**) Gene set analysis using the hallmark gene sets. The dot describes the enrichment (red) or depletion (green) of each hallmark gene set.

In addition, we performed a differential proteomic analysis between normal pancreatic cells and every other pancreatic cancer cell type to study pancreatic cancer-related pathways. AsPC-1, BxPC-3, CFPAC-1, HPAC, PANC-1, and HPSC showed 2630, 2124, 2519, 2724, 2013, and 2193 proteins with significantly different expression levels, respectively (log_2_ FC = 2, p < 0.05) (Fig. 4a). Subsequently, we functionally annotated these differentially expressed proteins via signalling pathway analyses (p < 0.05). By manually annotating clustering results, we discovered the characteristics of the pancreatic cancer signalling pathway inherent in each cell line. In CFPAC-1 cells, the p53 signalling pathway was significantly inhibited, and ECM-receptor interaction, the NOD-like receptor signalling pathway, the AGE-RAGE signalling pathway in diabetic complications, and steroid hormone biosynthesis were significantly activated (Fig. 4b). In PANC-1 cells, O-glycan biosynthesis, nicotinate and nicotinamide metabolism, NOD-like receptor ECM-receptor interaction, and NF-κB signalling pathway were significantly activated (Fig. 4c). In AsPC-1 cells, signalling pathways such as AMPK, PI3K-Akt, Toll-like receptor, glycolysis/gluconeogenesis, and ECM-receptor interaction were significantly enriched (Fig. s2a). In BxPC-3 cells, the NF-κB, TNF, MAPK, and mucin-type O-glycan biosynthesis signalling pathways were significantly enriched (Fig. s2b). In HPAC cells, the cell cycle, pyrimidine metabolism, p53 signalling pathway, and drug metabolism-other enzyme signalling pathways were significantly inhibited, while fatty acid degradation, steroid biosynthesis, and adherent junction signalling pathways were significantly activated (Fig. s2c). In the pancreatic stellate cell line HPSC, the PI3K-Akt, EGFR tyrosine kinase inhibitor resistance, apoptosis, and endocrine resistance signalling pathways were significantly activated, while the ECM-receptor interaction was significantly inhibited (Fig. s2d).

Furthermore, the gene set analysis (GSA) of the hallmark gene set was used to analyse the molecular characteristics of each pancreatic cancer cell line (Fig. 4d). Gene set enrichment of allograft rejection, IL6 JAK STAT3 signalling, inflammatory response, and interferon-alpha response gene set enrichment were consistent in five pancreatic cancer cell lines. However, pancreatic stellate cells have different molecular characteristics from pancreatic cancer cell lines. A large number of studies have found that a variety of signalling pathways is involved in the EMT process of tumour cells, such as Wnt, transforming growth factor-β (TGF-β), Notch, (phosphatidylinositol-3-kinases, PI3K), and Hedgehog, which all were identified in the GSEA. The enrichment analysis results further show that EMT is closely related to the occurrence of pancreatic cancer.

## Usage Notes

### Sensitivity of GEM in cell lines of the library

**Fig. 5:**
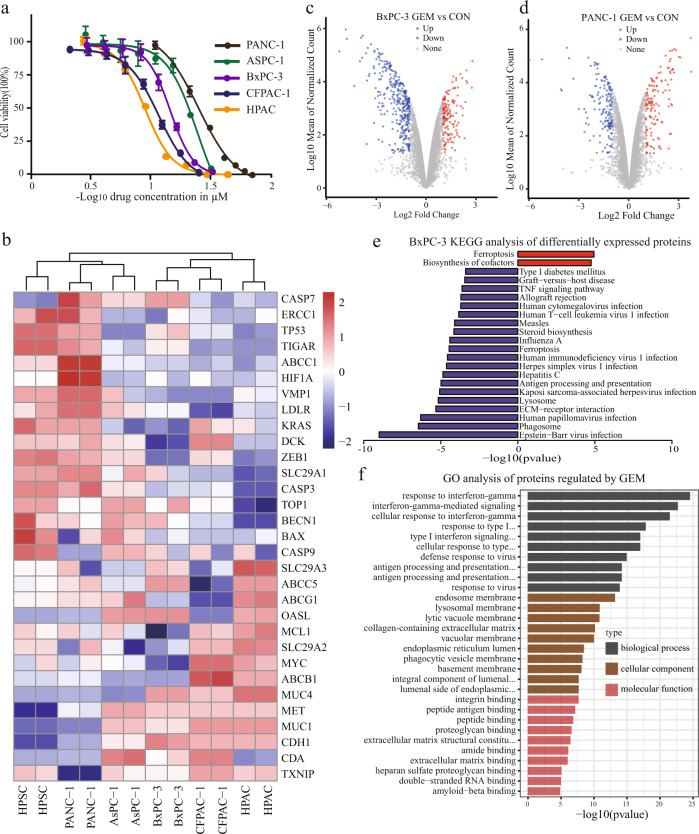
Sensitivity analysis of pancreatic cancer cell lines upon GEM treatment. (**a**) Dose-dependent inhibition of cell viability in pancreatic cancer cells. IC_50_ values were then determined using nonlinear regression using GraphPad Prism software. (**b**) Unsupervised clustering analysis of known GEM targets. Highly expressed genes are in red, and those with lower expression are in blue. (**c**,**d**) Volcano plot of differentially expressed proteins of GEM-treated BxPC-3 and PANC-1 cells. (**e**) KEGG analysis of differentially expressed proteins of BxPC-3 cells treated with GEM. (**f**) Gene ontology (GO) analysis of GEM-regulated proteins in BxPC-3 cells.

With the development of molecular biology, the regulatory mechanism of the metabolism and signalling pathways of GEM in pancreatic cancer cells has been revealed. Further research on these cellular and molecular mechanisms will help develop new chemotherapeutic sensitisation strategies and improve the effective rate of GEM chemotherapy, thereby enhancing the overall prognosis. *In vitro* cell line models have become one of the most important tools in pancreatic cancer research. Therefore, it is essential to investigate the sensitivity of GEM and the molecular biological mechanisms in different cell lines. In this study, a comprehensive proteomic analysis provided a valuable resource to study the GEM sensitivity of different pancreatic cancer cell lines. To investigate the effect of GEM on pancreatic cancer cell lines, we incubated the cell lines with different concentrations of GEM for 48 h and measured the cell growth using a CCK-8 kit. We observed that the cell growth in HPAC, CFPAC-1, BXPC-3, AsPC-1, and PANC-1 were dose-dependently inhibited, and the respective half-inhibitory concentration (IC_50_) was 13.57, 14.38, 19.56, 23.26, and 23.9 µM/L (Fig. 5a).

Based on the known GEM target genes and their functions, we evaluated the sensitivity of several cell lines to GEM, providing a colour-coded indication of the standardised gene expression (Fig. 5b). Genes such as BAX, ABCG1, MUC1, and CDH1 were overexpressed in HPAC, CFPAC-1, and BxPC-3 cell lines, indicating that GEM leads to tumour cell death by inducing endogenous apoptotic pathways. However, the expression of these genes was low in PANC-1, AsPC-1 and normal pancreatic cells. Studies have confirmed that the pro-apoptotic gene BAX is significantly downregulated in pancreatic cancer cells with GEM acquired resistance and that the ratio of BAX/BCL-2 can predict GEM sensitivity. The balance between pro-apoptotic and anti-apoptotic genes in the BCL-2 family determines the sensitivity of pancreatic cancer cells to GEM^38,39^. In PANC-1 and AsPC-1 pancreatic cancer cell lines, TOP1, BECN1, ERCC1, and other genes were overexpressed, compared to HPAC and normal cell lines. Patients in the high excision repair cross-complementation group 1 (ERCC1) tend to have a better overall survival rate as ERCC1 can repair GEM-induced chain breaks^40^. The induction of topoisomerase 1 (TOP-1)-mediated DNA breaks may contribute to GEM cytotoxicity^41,42^.

### Application of spectral library to DIA data

Evaluated performance of the library by exploring the potential GEM targets using the DIA strategy based on the spectral library. We performed proteomic analysis of BxPC-3 and PANC-1 cells before and after GEM treatment. DIA raw data files were searched against the spectral library using Spectronaut^TM^ with default settings. As a result, a total of 77928 unique peptides and 7089 corresponding proteins were identified, of which 712 were identified as differentially expressed proteins (log_2_ FC = 1, p < 0.05) (Fig. 5c,d). The potential GEM targets identified were differentially expressed in pancreatic cancer cell lines, indicating that the six cell lines had different degrees of GEM sensitivity (Fig. s3b). GO analysis of these differentially expressed proteins showed that most of them were components of the lysosomal membrane and collagen-containing extracellular matrix, primarily involved in the negative regulation of cytokine production, associated with the interferon-gamma-mediated signalling pathway, antigen processing, and presentation of endogenous peptide antigen biological processes (Fig. 5f, Fig. s3d). Furthermore, the KEGG pathway enrichment analysis showed that ferroptosis, lysosomes, and steroid biosynthesis were significantly inhibited (Fig. 5e, Fig. s3c). In addition, most ferroptosis-related proteins, such as ACSL1, SLC11A2, SLC39A8, STEAP3, TFRC, and TP53, were significantly downregulated by GEM. Additionally, the lysosomal-related proteins CYP51A1, FDFT1, LSS, MSMO1, and SQLE were significantly downregulated.

### The EMT phenotype of drug resistance in cell lines

**Fig. 6:**
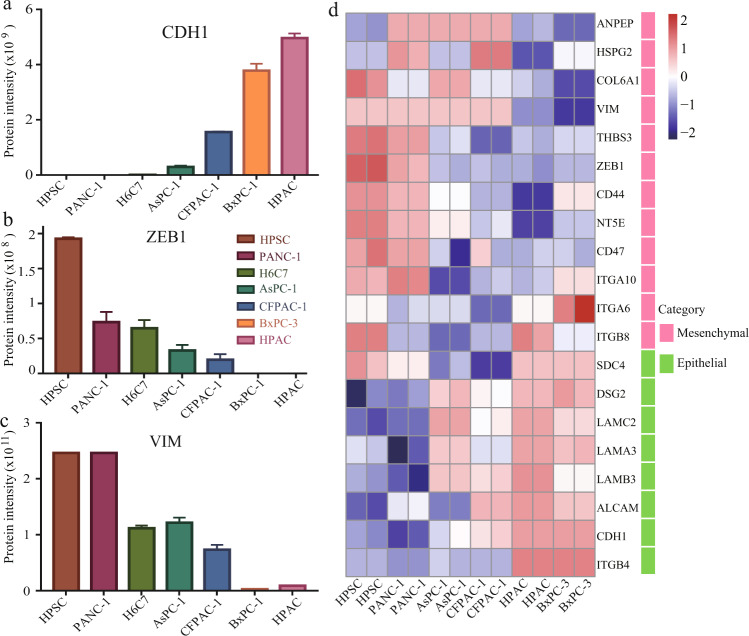
Analysis of drug resistance phenotypes. (**a**) Expression of EMT marker CDH1 in pancreatic cancer cell lines. (**b**) Expression of EMT marker ZEB1 in pancreatic cancer cell lines. (**c**) Expression of EMT marker VIM in pancreatic cancer cell lines. (**d**) Heat map of EMT targets for pancreatic cancer cell lines.

EMT is a dynamic process of cell transformation from an epithelial to a more aggressive mesenchymal type, which is pivotal for the invasion and metastasis of pancreatic cancer^43–45^. Numerous studies have suggested that EMT contributes to chemotherapy resistance in cancer cells^46,47^. Downregulation of proteins involved in cell junctions (such as E-cadherin and cytokeratin) and upregulation of mesenchymal molecular markers (such as fibronectin, vimentin, and N-cadherin) are hallmarks of the EMT phenotype^48,49^. Pathway enrichment analysis showed that the NF-κB, PI3K-Akt, VEGF, and ECM-receptor interaction pathways were significantly enriched in the AsPC-1, CFPAC-1, and PANC-1 cell lines. The epithelial cell marker CDH1 was significantly downregulated, whereas the mesenchymal cell markers VIM and ZEB1 were significantly upregulated (Fig. 6a–c). The proteins upregulated in BxPC-3 and HPAC cells were enriched in adherens junction, steroid biosynthesis, and mucin-type O-glycan biosynthesis, showing more epithelial properties. The signalling pathways NF-kappa B, MAPK, PI3K-Akt, and Toll-like receptors, which regulate EMT, were significantly enriched in the HPSC cell line. The GEM sensitivity of HPSC shown in Fig. 3b is similar to that of PANC-1. We then evaluated the expression of known EMT markers and found significant differences in the EMT biomarker characteristics of mesenchymal and epithelial cells (Fig. 6d).

Targeted inhibition of EMT has become a new strategy for overcoming GEM resistance in pancreatic cancer. The histone deacetylase (HDAC) inhibitor, mocetinostat, inhibits the expression of EMT-TF and ZEB1 by restoring miR-203, rendering pancreatic cancer cells with an EMT phenotype sensitive to chemotherapy^50^. El Amrani *et al*.^51^ found that pancreatic cancer acquired drug resistance was only found in GEM-induced mesenchymal cells, mediated by the ERK/pathway. Inhibition of ERK1/2 phosphorylation or ZEB-1 expression decreased chemical resistance and increased GEM sensitivity. Namba *et al*.^52^ reported that EMT driven by the Akt/GSK3β/Snail1 pathway leads to acquired GEM resistance in pancreatic cancer cells. The antiviral drug zidovudine inhibits these signal pathways and restores GEM sensitivity. EMT inhibitors can enhance the chemosensitivity of drug-resistant tumour cells and have been applied in clinical research combined with standard chemotherapy or targeted therapy, such as disulfiram combined with GEM, for metastatic pancreatic cancer (www.clinicaltrials.gov). In conclusion, changes in the EMT status are closely related to GEM resistance. Different pancreatic cancer mesenchymal cell characteristics may lead to different results. Future research should focus on the stratification of pancreatic cancer according to heterogeneity and the development of more accurate GEM sensitisation programmes.

## Supplementary information

Pancreatic cancer cells spectral library by DIA-MS and the phenotype analysis of gemcitabine sensitivity

## Data Availability

No custom computer codes were generated in this work.
