# Supplementary material for: Pancreatic cancer cells spectral library by DIA-MS and the phenotype analysis of gemcitabine sensitivity
Source: Sci Data. 2022 Jun 9;9:283. doi: 10.1038/s41597-022-01407-1 (PMC9184632; doi:10.1038/s41597-022-01407-1)
Supplement: Supplementary file 1 — Pancreatic cancer cells spectral library by DIA-MS and the phenotype analysis of gemcitabine sensitivity [file 41597_2022_1407_MOESM1_ESM.docx]

**Supporting Information:**

**Pancreatic cancer cells spectral library by DIA-MS and the phenotype analysis of gemcitabine sensitivity**

**Authors**

Ran Kong, Xiaohong Qian*, Wantao Ying*

**Affiliations**

State Key Laboratory of Proteomics, Beijing Proteome Research Center, National Center for Protein Sciences (Beijing), Beijing Institute of Lifeomics, Beijing, China

corresponding authors:

Xiaohong Qian (e-mail: qianxh1@163.com)

Wantao Ying (e-mail: yingwantao@ncpsb.org.cn.)


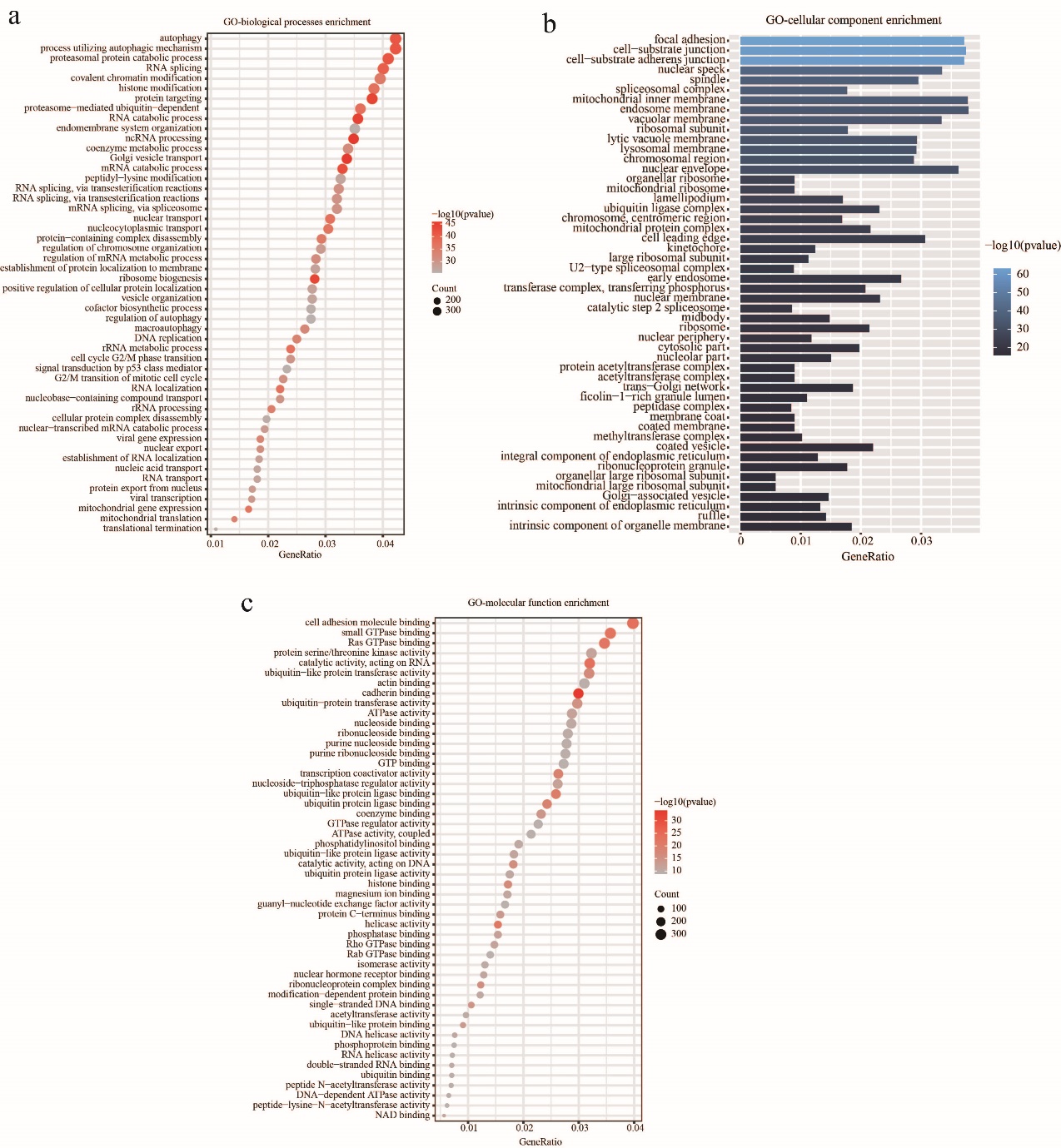


Supplementary Fig. s1: Gene ontology (GO) enrichment for all proteins identified in the DIA spectral library. (a). Dot plot of biological processes enrichment. Gene ratio is calculated as the number of proteins in the DIA spectral library against all genes with the same GO term. (b). Bar plot of cellular component enrichment. Length of bar indicates the number of proteins. (c). Dot plot of molecular function enrichment.


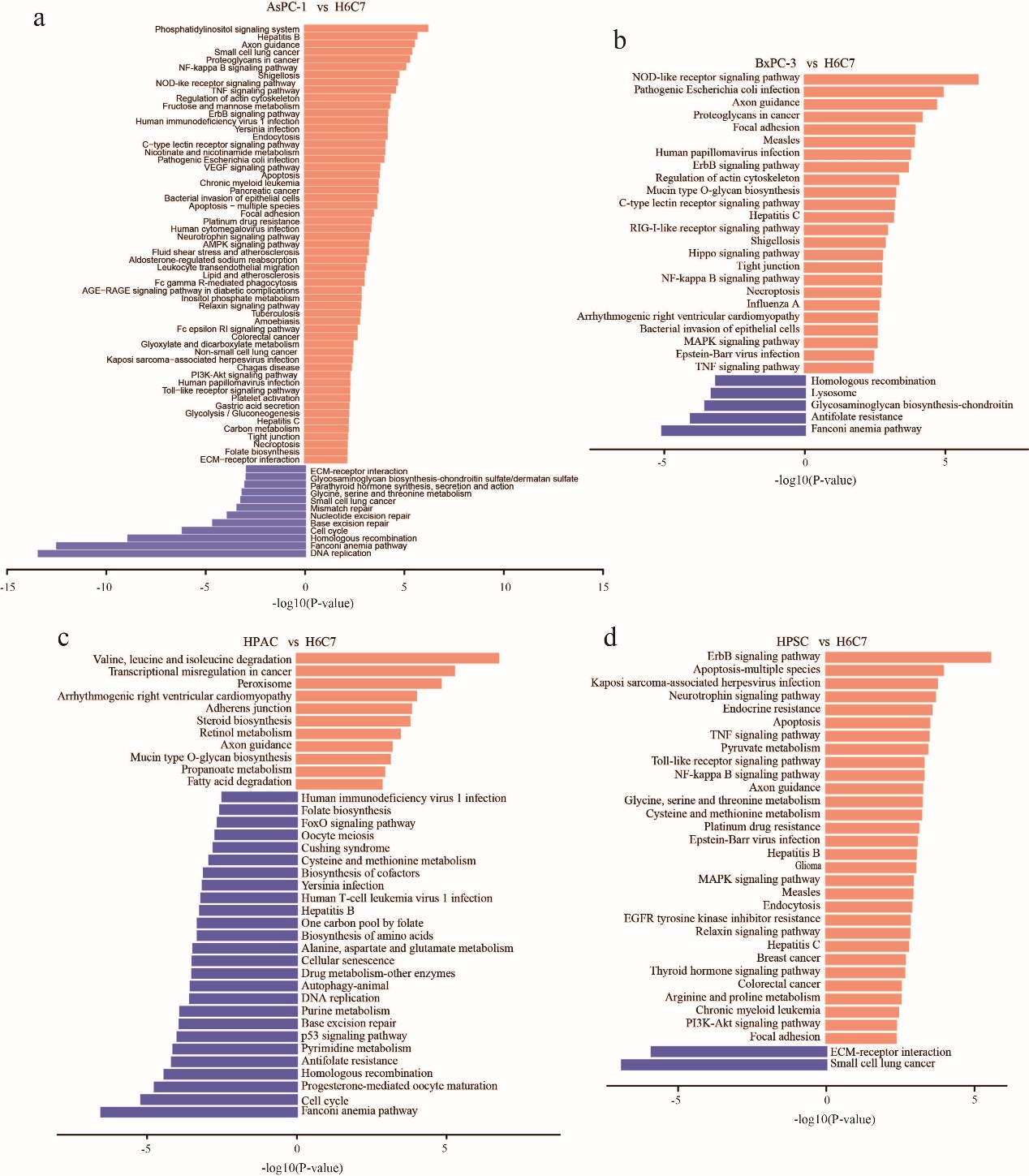


Supplementary Fig. s2: Analysis of pathways related to pancreatic cancer cell lines. (a) AsPC-1 vs H6C7 KEGG analysis of differentially expressed proteins. (b) BxPC-3 vs H6C7 KEGG analysis of differentially expressed proteins. (c) HPAC vs H6C7 KEGG analysis of differentially expressed proteins. (d) HPSC vs H6C7 KEGG analysis of differentially expressed proteins.


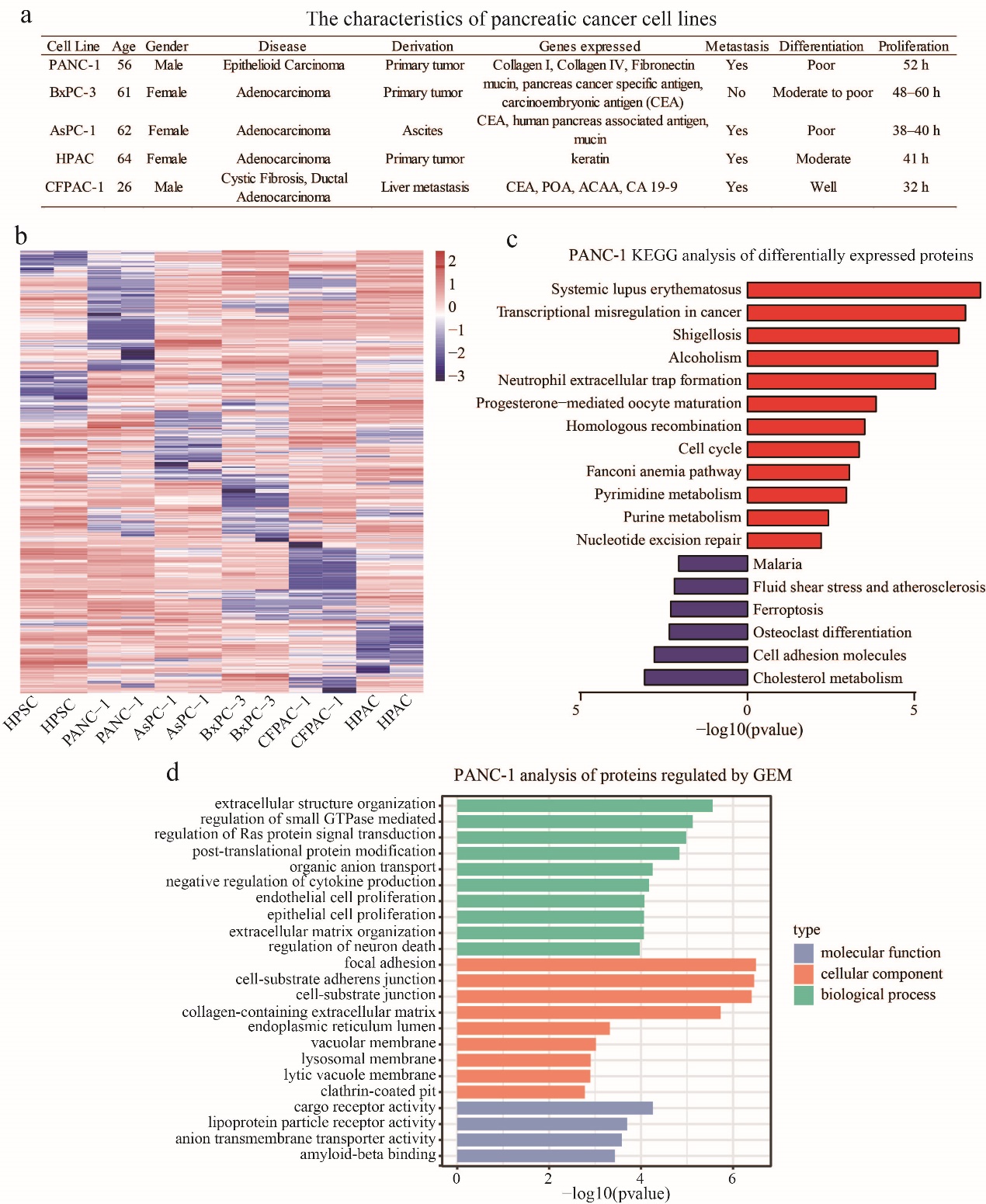


Supplementary Fig. s3: GEM related differentially expressed proteins. (a) The characteristics of pancreatic cancer cell lines. (b) Expression of potential GEM targets in pancreatic cancer cell lines. (c) KEGG analysis of differentially expressed proteins of PANC-1 cells treated with GEM. (d) Gene ontology (GO) analysis of proteins regulated by GEM in PANC-1.
